# Factors affecting the efficiency of *Rhizobium rhizogenes* root transformation of the root parasitic plant *Triphysaria versicolor* and its host *Arabidopsis thaliana*

**DOI:** 10.1186/s13007-018-0327-2

**Published:** 2018-07-16

**Authors:** Pradeepa C. G. Bandaranayake, John I. Yoder

**Affiliations:** 1 Agricultural Biotechnology Centre, Faculty of Agriculture, University of Peradeniya, Peradeniya, 20400 Sri Lanka; 2 Department of Plant Science, University of California Davis, Davis, CA USA

**Keywords:** *Rhizobium rhizogenes*, Parasitic plants, Orobanchaceae, Hairy roots, Root transformation

## Abstract

**Background:**

*Rhizobium rhizogenes* transformation is commonly used to generate transgenic roots traditionally called hairy roots, for both investigative and commercial applications. While fertile plants can be regenerated from transgenic roots, the transgenic roots are more typically used directly, either to investigate root biology or to produce valuable secondary metabolites. Hairy roots have been particularly useful for genetic studies of rhizosphere interactions; including the recognition of host plant roots by the roots of parasitic angiosperms.

**Results:**

In this manuscript we analyzed various environmental, nutritional and procedural conditions for their effects on transformation of the model hemi-parasitic plant *Triphysaria versicolor* and *Arabidopsis thaliana*, one of its hosts. We first examined the effects of media, gelling agents and co-incubation times on *Triphysaria* root transformation and determined that while all three affected transformation rates, the media were the most significant. Once those primary conditions were fixed, we examined the roles of seedling age, explant type, acetosyringone, temperature and illumination on *Triphysaria* hairy root transformation rates. Using the optimized procedure approximately 70% of *Triphysaria* seedlings developed transgenic roots as judged by expression of YFP. These conditions were then used to transform *Arabidopsis* and similar transformation rates were obtained.

**Conclusions:**

Analyses of root transformation factors provides a method recovering transgenic roots from both parasitic plants and their hosts at high frequency. In addition to providing an effective in vitro approach for genetic investigations of parasitic plant-host plant interactions, these results are applicable to genetic studies of non-parasitic plants as well.

**Electronic supplementary material:**

The online version of this article (10.1186/s13007-018-0327-2) contains supplementary material, which is available to authorized users.

## Background

*Rhizobium rhizogenes* (*previously Agrobacterium rhizogenes* [1]) is a soil born bacterium that infects dicotyledonous plants. The bacterium is chemotactically attracted to the plants by recognizing phenolic molecules, notably acetosyringone, that are released by wounded plants tissues [2]. When *R. rhizogenes* contacts the wound site, it transfers and integrates a piece of its Root inducing (Ri) plasmid bearing root locus (*rol*) genes into the plant genome, resulting in the proliferation of multi branched, hairy roots at the site of infection [3]. The transferred DNA encodes enzymes necessary for stimulating hairy root proliferation and, as has been done with *Agrobacterium tumefaciens*, provides a shuttle for introducing foreign genes into plants.

*Rhizobium rhizogenes* transformed roots can be regenerated into mature fertile transgenic plants [4], but more commonly transgenic root cultures are used directly to produce desirable compounds or to study gene function [5, 6]. Root cultures provide a useful means for producing desirable molecules that are naturally made by plants and secondary metabolites have been isolated from hairy roots of over 150 plant species [7]. For examples, the antimalarial drug artemisinin can be recovered from root cultures of *Artemisia annua* [8] and morphinan alkaloids can be recovered from cultures of *Papaver orientale* [9]. *R. rhizogenes* is also used to transform foreign genes into roots in order to produce novel products not present in the targeted host plant, such as *Nicotiana benthamiana* roots that produce a tumor-targeting therapeutic antibody with a glycosylation profile similar to that of humans [10].

In addition to metabolite production, transgenic roots have been extremely useful for investigating the biology of plant roots [11]. This is particularly useful for investigations of root specific phenotypes where regeneration of mature, fertile plants is not possible or otherwise impractical [12]. Transforming hairpin constructions that encode inhibitory RNAs have extended the utility of hairy root transformations [5, 13] and recently an atlas of tomato root tissues has been developed using *R. rhizogenes* [14]. The ability to directly assay transgenic roots has been very useful to investigate the interaction of roots with other organisms, including nematodes [15], nitrogen fixing rhizobia [16], arbuscular mycorrhiza [17, 18], and parasitic plants, in particular root parasitic plants in the Orobanchaceae [19–21].

Parasites in the Orobanchaceae directly invade the roots of host plants to rob them of nutritional resources. The effects on the host can be debilitating and parasitic weeds are a significant threat to agriculture internationally [22]. Parasitic plants attach to and invade host roots via parasite-encoded haustoria that develop in response to chemical and tactile cues from the host root and the development of haustoria and their infection into a host root can be monitored in vitro [23, 24]. We previously demonstrated that *R. rhizogenes* transformed *Triphysaria* roots form haustoria upon treatment with haustorial inducing factors and that these haustoria are functional and able to transport regulatory, hairpin generated RNAi molecules from host to parasite [25–27]. Similar results have been reported for the parasitic plant *Phtheirospermum japonicum* [20, 28].

The ability to generate transgenic roots of both host and parasitic plants has proven hugely advantageous for parasitic plant-host plant investigations [26–31]. However the frequency of root transformation using these procedures is rather low with only about 10–20% of seedlings yielding transgenic roots [19, 20]. In order to effectively exploit parasitic plant root transformation to explore the function of large numbers of candidate genes identified through genomic approaches, a more efficient transformation procedure is necessary. In this paper we examined several factors that affect root transformation of two species, *Triphysaria versicolor*, a model hemiparasitic species for parasitic plant investigations [32], and *Arabidopsis thaliana*, one of its hosts [33]. Under the optimized conditions about 70% of both *Triphysaria* and *Arabidopsis* seedlings developed transgenic roots. The ability to generate transgenic roots at high frequencies provides a rapid and effective means to manipulate both the host and parasitic partners in this root–root interaction.

## Results and discussion

### *Triphysaria* transformation: the effects of medium, solidifying agent and length of co-incubation

A three factor experiment was performed to evaluate the relative merits of co-incubating the seedlings and bacterial cultures in different media (MS or 0.25X Hoagland’s), different gelling agents (Phytagel or Phyto agar), and for different co-incubation periods at 16 °C (1 or 2 weeks).

As seen in Table 1, transformation efficiencies were significantly associated with all three factors. Since the interactions among the factors were significant, the specific effect of each factor on the transformation efficiency was evaluated. The MS medium resulted in higher transformation efficiencies than Hoagland’s regardless of solidifying agent or co-incubation times (Table 1, Additional file 1: Fig. S1A–B). Phytagel led to higher transformation rates than Phyto agar in both media and co-incubation periods (Additional file 1: Fig. S1C–D). Two weeks of co-incubation was significantly better than 1 week in MS medium but the time of co-incubation had no significant effect in Hoagland’s medium (Additional file 1: Fig. S1E–F).

**Table 1:** Effects of medium, solidifying agent and co-incubation on *Triphysaria* root transformation

Medium	Solidifying agent	Co-incubation period	% of YFP calli (after 4 weeks)	% Transformation (after 8 weeks)
MS	Phytagel	1 week	36.8±5	24.4±1
		2 weeks	75.9±12	70.0±10
	Phyto agar	1 week	23.9±2	19.1±2
		2 weeks	49.4±8	41.7±9
Hoagland	Phytagel	1 week	14.0±5	8.4±3
		2 weeks	12.2±2	8.7±2
	Phyto agar	1 week	8.6±1	6.2±2
		2 weeks	7.6±1	4.8±2
Medium (M) Pr > F			< 0.0001	< 0.0001
Solidifying agent (S) Pr > F			< 0.0001	0.0002
Duration (D) Pr > F			< .00001	< 0.0001
S*D Pr > F			0.0495	< 0.0088
M*S Pr > F			0.0014	< 0.0043
M*D Pr > F			< 0.0001	< 0.0001
M*S*D Pr > F			0.0358	0.0209

Each treatment consisted of five plates (each plate is a technical replicate) and the experiments were conducted three times (biological replicates). The efficiency values represent the average ± SD (n = 15)

Three weeks of co-incubation did not further increase transformation rates (Table 2). Co-incubation in dark has positive impact on transformation efficiency in some species [34]. However for *Triphysaria*, co-incubation in 24 h darkness resulted in fewer YFP expressing roots than maintaining the plants in a 12 h light/dark cycle (Table 2).

**Table 2:** Effects of biological and environmental parameters on *Triphysaria* root transformation

Parameter tested	Levels	Transformation efficiency	Statistics
Sugar content in the medium	0%	2.26 ± 0.8^c^	Pr > F < 0.0001
	0.5%	11.74 ± 5.0^bc^	
	1%	20.84 ± 5.3^b^	
	3%	73.93 ± 9.0^a^	
	4%	74.07 ± 6.0^a^	
Age of the seedling	5 days	67.91 ± 4.1^a^	Pr > F < 0.0013
	10 days	54.18 ± 2.4^b^	
	15 days	37.31 ± 8.1^c^	
Acetosyringone	0	3.25 ± 0.9^c^	Pr > F < 0.0001
	200 uM	21.74 ± 4.5^b^	n = 366–376
	400 uM	65.03 ± 6.4^a^	
Days kept in the co-incubation plate	7 days	33.21 ± 8.5^c^	Pr > F < 0.0008
	14 days	68.33 ± 4.2^a^	
	21 days	61.26 ± 4.1^a^	
Explant type	Hypocotyl	67.20 ± 2.8^a^	Pr > |t| < 0.0001
	Root tip	0.76 ± 0.8^b^	
Illumination during co-incubation	Light	67.53 ± 6.7^a^	Pr > |t| < 0.0001
	Dark	12.46 ± 2.6^b^	

Each treatment consisted of three plates (each plate is a technical replicate) and the experiments were conducted three times (biological replicates). The efficiency values represent the average ± SD (n = 09). Mean transformation efficiency, of a parameter tested, represented by different letters are significantly different at the *P* value given

The inclusion of 3% sucrose in the media increased transformation rates significantly (Table 2). Transformation rates were not further increasing in media with 4% sucrose and *R. rhizogenes* more rapidly overgrew the seedlings, therefor 3% sucrose was considered optimal.

Acetosyringone added to the media to induce the *vir* genes in *R. rhizogenes* and increase transformation rates. We assayed three concentrations of acetosyringone, 0, 200 and 400 µM, freshly added into the co-incubation plates. Increasing concentrations of acetosyringone were beneficial for transformation and its absence resulted in very low transformation rates (3%) (Table 2).

The age at which the seedlings were inoculated with MSU440 affected transformation rates with younger explants being more successful than older (Table 2). In some systems, inoculating root tips produced higher transformation rates than inoculating hypocotyls [17]. But co-incubating *Triphysaria* root tips with *R. rhizogenes* produced very few YFP expressing roots (Table 2).

### Effect of culture medium and temperature during co-incubation and subsequent root growth

A recurring problem affecting hairy root transformation is the overgrowth of seedlings with *R. rhizogenes*. Effective transformation is a balance of conditions that allow for the bacteria to infect and transform the seedlings but not overgrow the seedlings and media. The co-incubation conditions relevant to bacterial overgrowth include media, temperature and time of co-incubation.

Bacterial overgrowth was particularly problematic in Hoagland’s mediums and MSU440 could overgrow *Triphysaria* seedlings after 3–5 days. In contrast, MSU440 did not overgrow seedlings in MS medium until 12–14 days of co-incubation. The rapid growth of *R. rhizogenes* following co-incubation in Hoagland’s was a key factor in the reduced transformation rate compared to MS medium. However 0.25X Hoagland’s is a better medium for *Triphysaria* growth [25]. Therefore, we evaluated the effect of co-incubating the seedlings with MS for 2 weeks and then transferring them into either fresh MS medium or 0.25X Hoagland’s. After 8 weeks, there were a similar number of transgenic calli in both media but the total number of roots, their lengths and ultimately the transformation frequencies, were higher after transferring to Hoagland’s media than keeping the plants in MS after co-incubation (Table 3).

**Table 3:** Effects of culture medium on *Triphysaria* transformation and subsequent root growth

Measurement	Medium		Statistics
	0.25X Hoagland	MS	
% of YFP calli	73.26 ± 3.1^a^	75.21 ± 4.1^a^	Pr > |t| 0.5482
Transformation efficiency %	64.66 ± 4.4^a^	17.86 ± 9.3^b^	Pr > |t| 0.0014
Total number of roots	7.50 ± 0.3^a^	3.20 ± 0.1^b^	Pr > |t| < 0.0001
Number of YFP roots	2.00 ± 0.3^a^	0.25 ± 0.1^b^	Pr > |t| 0.0005
% of YFP roots	29.08 ± 4.8^a^	5.60 ± 1.6^b^	Pr > |t| 0.0013
Root length	2.67 ± 0.3^a^	1.91 ± 0.1^b^	Pr > |t| 0.0117

Each treatment consisted of three plates (each plate is a technical replicate) and the experiments were conducted three times (biological replicates). The efficiency values represent the average ± SD (n = 09). Mean transformation efficiency, of a measurement, represented by different letters are significantly different at the *P* value given

In our previously published method *Triphysaria* seedlings were shifted to 24 °C after co-incubation with *R. rhizogenes* at 16 °C. To test the effects of this temperature shift, seedlings were co-incubated with MSU440 at 16 °C for 2 weeks, transferred into fresh Hoagland’s plates and then incubated either 24 or 16 °C. While the total number of roots was higher and their growth rates faster when plants were shifted to 24 °C, the number of YFP roots per plant was the same as when maintained at 16 °C (Table 4). Therefore the transformation efficiency was significantly higher when *Triphysaria* were maintained at 16 °C following co-incubation (Table 4).

**Table 4:** Effects of temperature on transformation and subsequent growth of *Triphysaria* roots

Measurement	Time at 16 °C		Statistics
	2 weeks at 16 °C + 6 weeks in 25 °C	8 weeks at 16 °C	
Transformation efficiency (%)	28.25 ± 13.9^b^	62.36 ± 4.0^a^	Pr > |t| 0.0149
Total number of roots	13.23 ± 09^a^	6.87 ± 0.7^b^	Pr > |t| 0.0006
Number of YFP roots	1.15 ± 0.0^a^	1.52 ± 0.2^a^	Pr > |t| 0.1355
% YFP roots	9.81 ± 0.8^b^	24.58 ± 2.9^a^	Pr > |t| 0.0011
Length of YFP roots (cm)	3.30 ± 0.1^a^	2.31 ± 0.1^b^	Pr > |t| 0.0005

Each treatment consisted of three plates (each plate is a technical replicate) and the experiments were conducted three times (biological replicates). The efficiency values represent the average ± SD (n = 09). Mean transformation efficiency, of a measurement, represented by different letters are significantly different at the *P* value given

### Application of modified hairy root transformation to *Arabidopsis*

We next examined the effects of some of these modifications on *Arabidopsis* hairy root transformation, as previously described by Limpens et al. [5]. One of the most profound parameters affecting transformation rates was the age of the *Arabidopsis* seedling at inoculation; inoculating 2-day-old *Arabidopsis* seedlings resulted in about 70% of them having transgenic roots while the same protocol using 10-day-old seedlings barely produced any transgenic roots (Fig. 2). As with *Triphysaria*, younger seedlings resulted in higher transformation efficiencies.

We also evaluated *Arabidopsis* transformation efficiencies with different times of co-incubation at 16 °C and with or without a subsequent shift to 25 °C (Table 5). Unlike *Triphysaria*, *Arabidopsis* performed poorly with extended periods of time at 16 °C. Subsequent growth at low temperature also had a negative impact on transformation efficiency of *Arabidopsis.* The data suggest that there is an interaction between these two factors (Table 5).

**Table 5:** Effect of temperature and co-incubation period on *Arabidopsis* root transformation

Duration in co-transformation plate (days)	Growth temperature (°C)	Transformation efficiency (Mean ± SD)
3	25	72.05 ± 5.7^a^
	16	14.86 ± 3.4^b^
6	25	32.82 ± 9.2^a^
	16	6.40 ± 0.9^b^
9	25	13.56 ± 4.4^a^
	16	3.29 ± 1.5^b^
12	25	2.50 ± 2.4^a^
	16	0.49 ± 0.3^a^
Duration in 16 °C Pr > F 0.0001		
Growth temperature (°C) Pr > F 0.0001		
Duration in 16 °C * Growth temperature(°C) Pr > F 0.0001		

Each treatment consisted of five plates (each plate is a technical replicate) and the experiments were conducted three times (biological replicates). The efficiency values represent the average ± SD (n = 09). Significantly different transformation efficiencies are represented by different letters

**Fig. 1:**
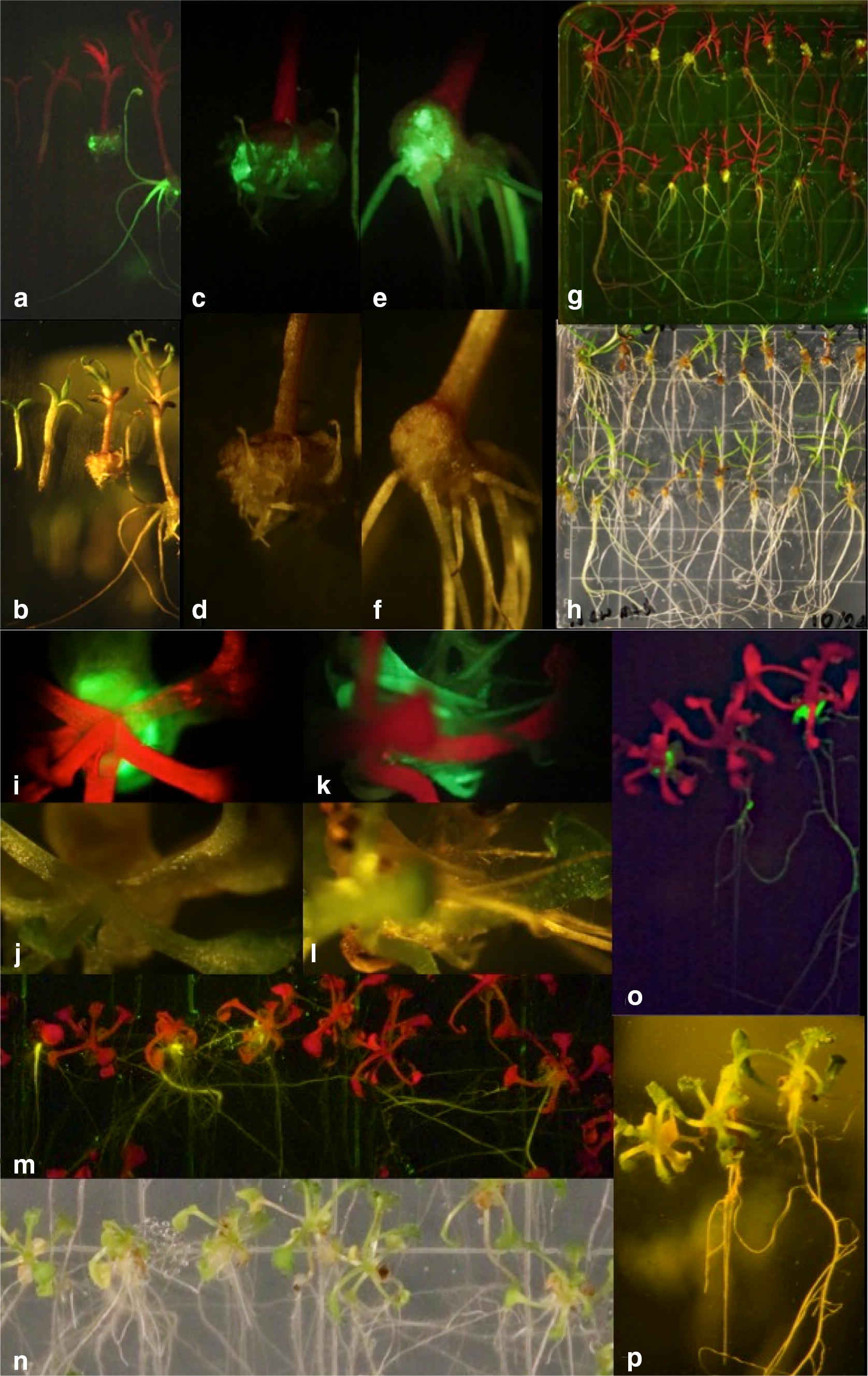
*Rhizobium rhizogenes* mediated root transformation of *Triphysaria* and *Arabidopsis,* **a**, **c**, **e**, **g**, **i**, **k**, **m**, **o** images under YFP florescence, **b**, **d**, **f**, **h**, **j**, **l**, **n**, **p** images under white light, **a**, **b** *Triphysaria* 0, 5, 14 and 60 days after inoculation with *R. rhizogenes,* **c**, **d** *Triphysaria* callus development 14 days after inoculation, **e**, **f** *Triphysaria* callus development 60 days after inoculation, **g**, **h** Overview of plate containing *Triphysaria* 60 days after inoculation, **i**, **j** *Arabidopsis* callus development 7 days after inoculation, **k**, **l** *Arabidopsis* callus development 14 days after inoculation, **m**, **n** overview of plate containing *Arabidopsis* 21 days after inoculation, **o**, **p** *Arabidopsis* 7, 14 and 21 days after inoculation

**Fig. 2:**
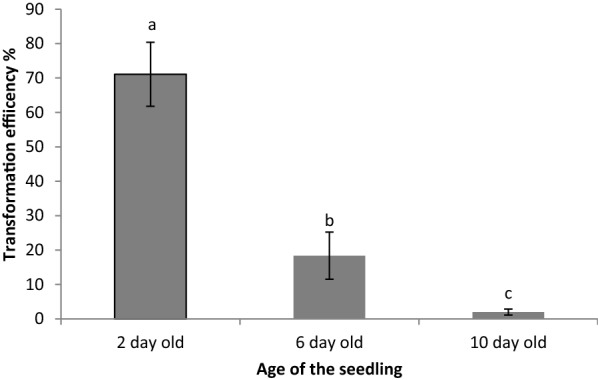
Effects of seedling age on *Arabidopsis* transformation efficiency, Each value represents mean ± SD of three transformation experiments each with 3 technical replicates. Values represented by different letters are significantly different (Pr > F = < 0.0001)

Under the optimized conditions, transgenic calli expressing YFP were apparent within 7 days of transformation (Fig. 1i, j) and YFP expressing roots appeared within 14 days (Fig. 2k, l). The *Arabidopsis* transformation efficiency reported here is considerably higher than that of the previously published method [5].

### PCR analysis of YFP expressing transgenic roots

We used PCR to confirm transformation. We used primers that selectively amplify genes present on the T-DNA and incorporated into the plant genome. We also used primers that selectively amplify vir genes as control against contaminating *R. rhizogenes* because vir genes do not integrate into the plant genome [35]. *Triphysaria TvQR1* gene and *Arabidopsis* actin gene were used as a positive control for amplification of plant cDNA.

**Fig. 3:**
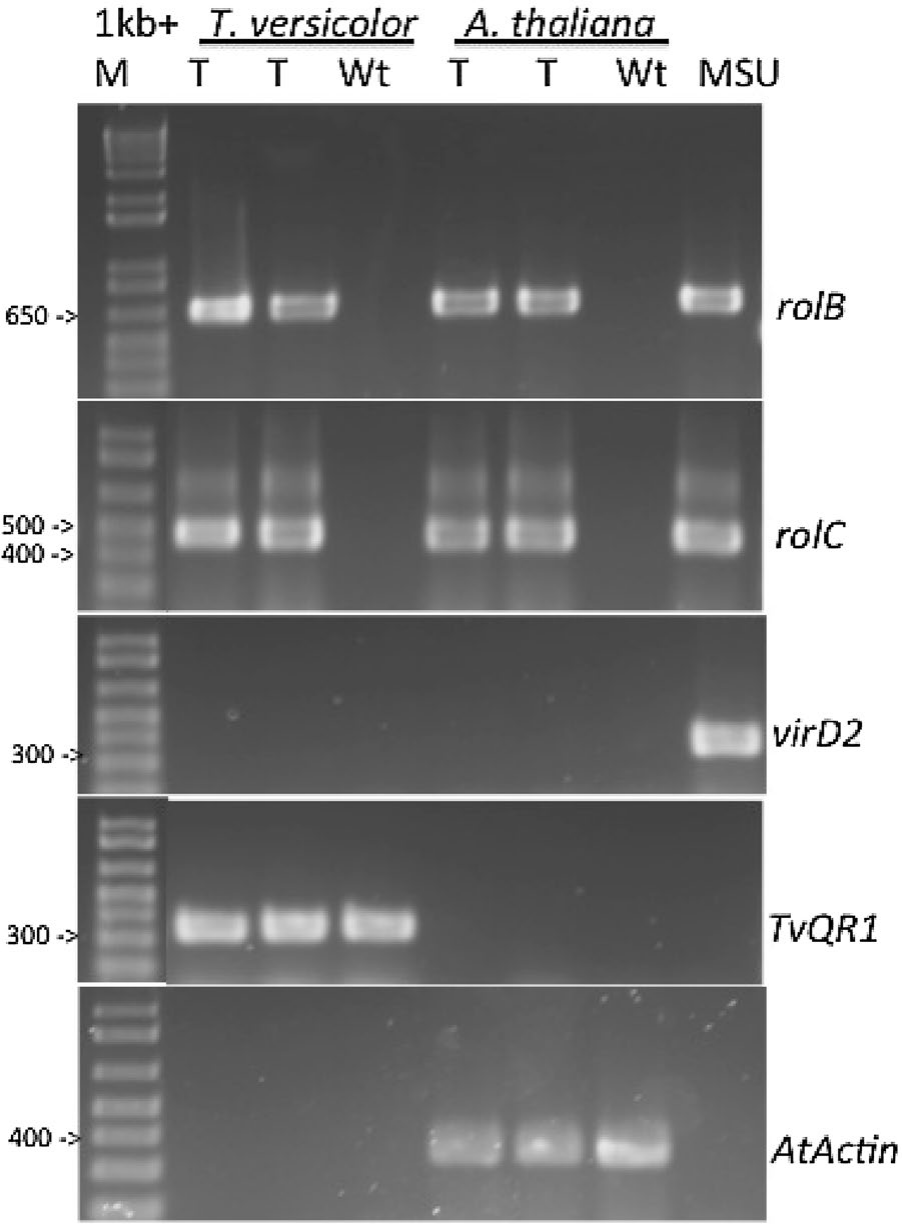
Confirmation of *R. rhizogenes* Ri plasmid integration in YFP expressing *T. versicolor* and *A. thaliana* roots. Primers for *rolB*, *rolC*, *virD2*, *TvQR1* and *AtActin* were used in PCR reactions using cDNA from transgenic (T) or non-transformed (Wt) roots. MSU is genomic DNA from MSU440 and M is the 1 Kb+ DNA ladder. The cDNA from wild type roots and genomic DNA from *R. rhizogenes* MSU440 were used as negative and positive controls. *T. versicolor* TvQR1 gene and *A. thaliana* Actin gene were used as a positive control for amplification plant cDNA. The *rolB* and *rolC* genes were amplified from hairy root cDNA. The *virD2*, an *R. rhizogenes* gene present on the Ri plasmid but not inserted into the plant genome, was amplified from YFP expressing roots

The cDNA from wild type roots and genomic DNA from *R. rhizogenes* MSU440 were used as negative and positive controls. Figure 3 shows that the *rolB* and *rolC* genes, but not *virD2*, were amplified from the YFP expressing transgenic roots, indicating that *rolB* and *rolC* are incorporated into the plant genome.

## Conclusions

This paper describes the optimization of *R. rhizogenes* transformation of the root parasitic plant *Triphysaria* and its host *Arabidopsis*. The two optimized procedures are slightly different and summarized below.

Five-day-old *Triphysaria* seedlings were inoculated with MSU440 and placed onto plates with MS medium, 3% w/v sucrose, 400uM acetosyringone and 0.2% Phytagel. Seedlings were co-incubated for 14 days at 16 °C at which time there were transferred to 0.25X Hoagland’s medium with 300 mg/L timentin, 0.75% sucrose and 0.8% Phyto agar. The freshly plated seedlings were returned to the 16 °C incubator and after 8 weeks about 60% of the *Triphysaria* seedlings had at least one YFP expressing root.

Two-day-old *Arabidopsis seedlings* were inoculated with MSU440 and placed onto plates with MS medium, 3% w/v sucrose, 400uM acetosyringone and 0.2% Phytagel. Seedlings were co-incubated for 3 days at 16 °C at which time there were transferred to 0.25X Hoagland’s medium with 300 mg/L timentin, 0.75% sucrose and 0.8% Phyto agar and incubated at 25 °C. About 70% of the *Arabidopsis* seedlings had one or more roots ready to assay with 21 days of transformation.

## Methods

### Plasmids, media, bacteria and plants

The R. *rhizogenes* strain MSU440 contains the root inducing plasmid pRiA4 [34]. MSU440 was transformed with the T-DNA based vector pHG8-YFP which expresses the Yellow Fluorescence Protein (YFP) as a visual marker for transformation [35].

*Triphysaria* versicolor seeds were collected from an open pollinated population growing south of Napa, California. *Arabidopsis thaliana* Columbia seeds were obtained from the *Arabidopsis* Biological Resource Center (https://abrc.osu.edu/).

Hoagland’s medium was used at quarter strength (0.25X) and was prepared in the lab. Murashige and Scoog medium with vitamins (M519) was purchased in powder form from Phytotechnology Laboratories (Shawnee Mission, Kansas) and used at full strength. The composition of both media are shown in Additional file 2: Table S1.

Media were solidified with either 0.2% Phytagel (Sigma) or 0.8% Phyto agar (Plant Media).

### *Rhizobium rhizogenes* mediated root transformation

Below are our optimized methods for hairy root transformation of *Triphysaria* and *Arabidopsis*. Several of these conditions were determined from the experiments described in this manuscript.

A single colony of MSU440 was grown overnight in 1 ml of MGL medium containing 100 µg ml^−1^ spectinomycin (Additional file 3: Table S2). The bacterial culture was poured onto the top of a fresh MGL agar plate containing spectinomycin and 400 µM acetosyringone. The plate was incubated overnight at 28 °C until a creamy bacterial lawn developed on the plate.

*Triphysaria* seeds were surface sterilized by treating with 70% ethanol for 10 min, 50% hypochlorite and 0.2% Triton X-100 for 30 min and washed eight times with sterile distilled water. Seeds were then plated on 0.25X Hoagland’s medium supplemented with 0.75% (w/v) sucrose and 0.6% (w/v) Phyto agar and vernalized at 4 °C for 3 days before being transferred to 16 °C to germinate under about 11 µmol s^−1^ m^−2^ photosynthetically active radiation (PAR).

*Arabidopsis* seeds were surface sterilized by shaking for 5 min in 2% hypochlorite (commercial bleach) followed by washing with sterile water. *Arabidopsis* seeds were plated on MS medium supplemented with 1% sucrose and 0.6% Phytoagar, vernalized at 4 °C for 3 days, and transferred to 25 °C to germinate 30–461 µmol s^−1^ m^−2^ PAR.

Roots of 5-day-old *Triphysaria* seedlings or 2-day-old *Arabidopsis* were excised by making a sharp cut with a scalpel at the root-shoot junction. The cut surface of the hypocotyl was inoculated by dipping onto the agar plate containing the lawn of MSU440. The inoculated seedlings were placed in three rows, each row containing 15 plants, on square petri dishes containing MS medium supplemented with 3% (w/v) sucrose, 0.2% Phytagel and 400 µM acetosyringone. The inoculated seedlings were co-incubated with MSU440 at 16 °C with 12 h light at 50–86 1 µmol s^−1^ m^−2^ PAR; *Triphysaria* seedlings were co-incubated for 2 weeks and the *Arabidopsis* seedlings were co-incubated for 3 days at 16 °C.

After co-incubation, both *Triphysaria* and *Arabidopsis* seedlings were transferred to fresh 0.25X Hoagland’s plates containing 0.75% sucrose and 300 mg/L timentin (Plantmedia, USA) to reduce *R. rhizogenes* growth. *Triphysaria* seedlings were returned to the 16 °C incubator but *Arabidopsis* seedlings were shifted to 25 °C for further root development.

In *Triphysaria*, transgenic calli were identified 4 weeks after co-incubation and transgenic roots 8 weeks after co-incubation by visualization of YFP fluorescence (Fig. 1a–h) under a Zeiss Stemi SV11 dissecting microscope furnished with an YFP filter set with excitation HQ500/20, dichroic beam splitter Q515LP, and emission HQ535/30. Images were captured with a mounted camera. In *Arabidopsis*, transgenic calli were identified within 7 days after transformation and transgenic roots in 14 days (Fig. 1i–p).

## Data analysis

The transformation efficiency was calculated as the number of plants per plate with at least one root expressing YFP. Each plate was a technical replicate and containing between 35 and 50 seedlings, and each experiment consisted with three plates. Each experiment was a biological replicate and repeated two additional times. Data were analyzed with either the General Liner Model (Proc GLM) or the *t* test using the statistical analysis software, SAS 9.2 (SAS Institute Cary, North Carolina).

### Transgene detection

Total RNA was extracted from roots using Qiagen RNAeasy mini kit including the in-column DNAase treatment. The quality and quantity of RNA was determined with a NanoDrop spectrophotometer and by electrophoresis through a 1% agarose gel. We reverse transcribed 0.5 µg RNA using the Superscript III First-Strand Synthesis System (Invitrogen, Carlsbad, CA) and oligo (dT)20 primers and used the cDNA as template for PCR.

Two sets of primers amplified sequences on the T-DNA that is transferred into the host; one set amplified a 625 bp fragment of the *R. rhizogenes* rolB gene and one a 422 bp fragment of the rolC gene. A third set of primers amplified a 338 bp fragment of the virD2 gene which is not transferred into the host to control for bacterial contamination. Primers specific for the *Triphysaria* QR1 gene and the *Arabidopsis* actin gene were used as controls for endogenous plant genes. Primer sequences are shown in Additional file 4: Table S3. All PCR reactions were performed with Taq DNA polymerase (Invitrogen) using 35 cycles of 94 °C for 90 s, 55 °C for 30 s, 72 °C for 90 s, followed by a 10 min extension at 72 °C.

## Additional files

**Additional file 1: Figure S1.** Contribution of individual factors on transformation efficiency. The contributions of individual factors on the efficiency was compared using GLM procedure sorted by each factor followed by Tukey’s Studentized Range (HSD) Test. A–B: Media combinations, C–D: Solidifying agent. E–F: Co-cultivation duration.

**Additional file 2: Table S1.** Plant growth media composition.

**Additional file 3: Table S2.** Bacterial growth medium (MGL) composition.

**Additional file 4: Table S3.** Sequences of primers used for PCR.
