# Supplementary material for: Factors affecting the efficiency of Rhizobium rhizogenes root transformation of the root parasitic plant Triphysaria versicolor and its host Arabidopsis thaliana
Source: Plant Methods. 2018 Jul 16;14:61. doi: 10.1186/s13007-018-0327-2 (PMC6048883; doi:10.1186/s13007-018-0327-2)
Supplement: Supplementary file 1 — Additional file 1: Figure S1. Contribution of individual factors on transformation efficiency. The contributions of individual factors on the efficiency was compared using GLM procedure sorted by each factor followed by Tukey’s Studentized Range (HSD) Test. A–B: Media combinations, C–D: Solidifying agent. E–F: Co-cultivation duration. [file 13007_2018_327_MOESM1_ESM.docx]

A

B

C

D

E

F

**Additional Figure 1: Contribution of individual factors on transformation efficiency**
