# Supplementary material for: Factors affecting the efficiency of Rhizobium rhizogenes root transformation of the root parasitic plant Triphysaria versicolor and its host Arabidopsis thaliana
Source: Plant Methods. 2018 Jul 16;14:61. doi: 10.1186/s13007-018-0327-2 (PMC6048883; doi:10.1186/s13007-018-0327-2)
Supplement: Supplementary file 2 — Additional file 2: Table S1. Plant growth media composition. [file 13007_2018_327_MOESM2_ESM.docx]

# **Additional file 2: Table S1. Plant growth media composition**

| Item | 0.25× Hoagland (mg/l) | 1× MS (mg/l) |
| --- | --- | --- |
| Calcium chloride anhydrous |  | 332.2 |
| Calcium nitrate | 205 |  |
| Ammonium nitrate |  | 1650 |
| Potasium nitrate | 126 | 1900 |
| Magnisium sulfate | 60 | 180.7 |
| Potassium phosphate monobasic | 34 | 170 |
| Ferrous sulfate • 7H_2_O | 0.62 | 27.8 |
| NaEDTA | 0.84 | 37.28 |
| Boric acid | 1.2 | 6.2 |
| Manganese chloride • 4H_2_O | 0.69 |  |
| Manganese sulfate • H_2_O |  | 16.9 |
| Cupric sulfate • 5H_2_O | 0.035 | 0.025 |
| Zinc sulfate • 7H_2_O | 0.071 | 8.6 |
| Molybdic acid (sodium salt) • 2H_2_O | 0.022 | 0.25 |
| Sodium chloride | 1.5 |  |
| Cobalt chloride • 6H_2_O | 0.000744 | 0.025 |
| Potassium iodide |  | 0.83 |
| pH at room temperature | 6.1 | 5.6 |
