# Supplementary material for: Factors affecting the efficiency of Rhizobium rhizogenes root transformation of the root parasitic plant Triphysaria versicolor and its host Arabidopsis thaliana
Source: Plant Methods. 2018 Jul 16;14:61. doi: 10.1186/s13007-018-0327-2 (PMC6048883; doi:10.1186/s13007-018-0327-2)
Supplement: Supplementary file 3 — Additional file 3: Table S2. Bacterial growth medium (MGL) composition. [file 13007_2018_327_MOESM3_ESM.docx]

# **Additional file 3: Table S2. Bacterial growth medium (MGL) composition**

| Item | Among per liter (g) |
| --- | --- |
| Mannitol | 5 |
| L-glutamic acid | 1 |
| K_2_HPO_4_ | 0.25 |
| NaCl | 0.1 |
| MgSO_4_.7H_2_O | 0.1 |
| Biotin | 0.001 |
| Tryptone | 5.0 |
| Yeast Extract | 2.5 |
| Bactoagar (only for solid medium) | 15 |
| pH at room temperature | pH 7.0 with 1M NaOH |
