# Supplementary material for: Factors affecting the efficiency of Rhizobium rhizogenes root transformation of the root parasitic plant Triphysaria versicolor and its host Arabidopsis thaliana
Source: Plant Methods. 2018 Jul 16;14:61. doi: 10.1186/s13007-018-0327-2 (PMC6048883; doi:10.1186/s13007-018-0327-2)
Supplement: Supplementary file 4 — Additional file 4: Table S3. Sequences of primers used for PCR. [file 13007_2018_327_MOESM4_ESM.docx]

# **Additional file 4: Table S3. Sequences of primers used for PCR**

| Gene | Forward Primer (5’-3’) | Reverse Primer (5’-3’) | Reference |
| --- | --- | --- | --- |
| *rolB* | CGAGGGGATCCGATTTGCTTT | GACGCCCTCCTCGCCTTCCT | [39] |
| *rolC* | TGTGACAAGCAGCGATGAGC | GATTGCAAACTTGCACTCGC | [40] |
| *virD2* | ATGCCCGATCGAGCTCAAGT | CCTGACCCAAACATCTCGGCT | [40] |
| *TvQR1* | TTCCCATTGCCGGCCTTACA | CGACGCACAATGAATAACCG | [34] |
| *AtActin* | TCCGGTGTCCGGAAGTTCTGTTC | CCGTCTTCGTTTGGTGATCTTAGG | [41] |
